# Nicotine-Mediated Alterations in Exosome Content: Implications for Stroke and Neurological Dysfunction

**DOI:** 10.3390/biom16030463

**Published:** 2026-03-19

**Authors:** Christopher Grahe, Richard D. Egleton, Nalini Santanam, Ji Chen Bihl

**Affiliations:** Joan C. Edwards School of Medicine, Marshall University, Huntington, WV 25701, USA; grahe@marshall.edu (C.G.); egleton@marshall.edu (R.D.E.);

**Keywords:** nicotine, smoking, exosomes, stroke, neurovascular dysfunction

## Abstract

Nicotine damages the cardiovascular system in a variety of ways, from promoting inflammation to causing oxidative stress to prompting unnecessary autophagy. The alteration to the nervous system that yields nicotine dependence further exacerbates the negative impact that nicotine use has on public health. Nicotine use has also been found to cause alterations in exosome content, especially miRNA. Conversely, exosomes have also had promising results as treatments for nicotine-mediated alterations in protein and miRNA levels. However, although nicotine has been shown to both alter exosome content and exacerbate stroke outcomes, the relationship between these two functions is poorly understood. This review examines multiple sources to compare available data. Several factors in nicotine’s effect on exosome content were thus found that imply a correlation. Also, exosome contents are not only a viable biomarker for multiple conditions, including ischemic brain damage and tobacco use, but they are also able to influence the cells of test subjects, both as a treatment for ischemic stroke and as a regulator of healthy brain function in stroke-free test subjects. Taken together, previous evidence suggests that nicotine-mediated alterations in neuronal exosome content have an effect on the progression of stroke in nicotine users.

## 1. Introduction

Nicotine causes damage to the cardiovascular system and alters the nervous system to create a nicotine dependence. In an effort to find effective treatments for the damage resulting from nicotine dependence, multiple studies have examined exosomes as novel biomarkers of cardiovascular and neurological dysfunction. It has been found that exosomes are able to carry cargo, cross the blood–brain barrier, and deliver cargo to key pathological areas in a wide range of conditions and injuries, making them ideal biomarkers for a wide range of cardiovascular and neurological ailments. This has continued to prove clinically relevant, as many studies in tissues ranging from liver and lung to heart and brain have found that nicotine causes changes in exosome content and function, which may promote the development of other conditions. However, the relationship between nicotine and exosomes with respect to ischemic stroke is still largely unclear; though many papers in the past have examined the correlation between nicotine and ischemic stroke, between exosomes and ischemic stroke, and between nicotine and exosomes, a unifying molecular mechanism between all three areas has yet to be determined. This paper will summarize current research proceedings on the topics of exosomes, nicotine, and ischemic stroke to assess the knowledge that is present and determine the direction of future research.

## 2. Exosomes Overview

Exosomes are a type of extracellular vesicle secreted by various cells to dispense proteins, RNAs, lipids, and various other cellular products into the bloodstream [1]. This dispensing of packages of cellular products has the purpose of either facilitating communication between cells or disposing of excess metabolic by-products. According to Alzahrani et al. [2], Exosomes typically range from 40 to 160 nm in diameter, with an average size of 100 nm, and they form through the double invagination of the plasma membrane and the creation of intracellular multivesicular bodies (MVBs) containing intraluminal vesicles (ILVs). Exosomes are released by various cells, ranging from neurons to immune cells to muscle cells, into the bloodstream, and the contents of said exosomes have the potential to stimulate cell-to-cell communication.

Exosomes are capable of both local and long-distance intercellular communication, transferring proteins [3], lipids, and RNAs [4] to cells through the bloodstream. One RNA type that has received a lot of focus in past research is Micro-RNA (miRNA), since miRNA-containing exosomes show promise as biomarkers of cellular diseases such as chemical toxicities, including the flavor-related toxicities associated with electronic cigarette use [5]. Since exosomes carry cargo that reflects the condition of the cell that released them, they also show promise as a diagnostic tool for all sorts of maladies, including neuropathic conditions such as alcoholic neurotoxicity and HIV-related encephalitis [6].

## 3. Nicotine and Exosomes

Nicotine is a drug that activates nicotinic acetylcholine receptors (nAChRs), particularly in the brain and central nervous system. These activations support the initiation and maintenance of smoking behaviors, but they also significantly alter protein expression in the cortex, leading to significantly altered downstream signaling [7]. When nAChRs on dopaminergic neurons in the ventral tegmental area (VTA) are stimulated by nicotine, dopamine is released into the shell of the nucleus accumbens (NAcc), reinforcing the use of nicotine with neuronal adaptations over the course of days to months [8] Repeated use causes nAChRs to desensitize, which is believed to contribute to the development of nicotine dependence [9]. Nicotine also crosses the blood–brain barrier and induces neurological adaptations at the level of individual cells and proteins, causing further complications [10]. For instance, in a cohort of a nicotine-mediated conditioned place preference (CPP) group, matrix metalloproteinase 9 (MMP-9) expression in the hippocampus was differentially elevated compared to control animals [11]. Administering a broad-spectrum MMP inhibitor successfully blocked CPP development, suggesting that MMP-9 may be involved in facilitating intracellular and extracellular events required for the synaptic plasticity underlying acquisition of nicotine-mediated CPP. MMP-9 inhibition by administration of exosomes from healthy rats also helped reduce leakage of the BBB in ischemic stroke models [12], suggesting that exosomes could also help reduce the development of nicotine-mediated CPP. As MMP-9 is an enzyme that breaks down matrix proteins essential for cell motility and replication [13], its role in CPP development may involve clearing a path in the hippocampus for new neuronal connections to form, in response to altered signaling induced by exposure to nicotine.

Exosomal RNA content in particular is a reliable biomarker for changes to brain function caused by several different types of nicotine exposure, including waterpipe, e-cigarettes, regular paper cigarettes, and a combination of any two of the above. One study found seven miRNAs that were differentially expressed in exosomes, mainly originating from the heart and lungs, between smokers and non-smokers: hsa-let-7a-5p, hsa-miR-21-5p, hsa-miR-29b-3p, hsa-let-7f-5p, hsa-miR-143-3p, hsa-miR-30a-5p, and hsa-let-7i-5p [14]. Meanwhile, another study found a nearly 4-fold increase in BNIP3L (Bcl2 interacting protein 3-like protein) and general upregulation of both endonuclease reverse transcriptase proteins and long noncoding RNAs from the RNA-binding family in exosomes derived from the blood plasma of human smokers [15]. Nicotine exposure also increased the size and biogenesis of brain-derived extracellular vesicles (BDEVs), more significantly in females [16], consistent with past research showing that endothelial and platelet-derived extracellular vesicle concentration was increased in healthy volunteers exposed to e-cigarettes [17]. Thus, exosomes from astrocytes and neurons can be extracted from the blood to assess any changes in brain function caused by nicotine exposure [6].

E-cigarettes (also called vapes) in particular have had a lot of research done to understand them since their rise in popularity in 2018 [18]. The findings of one team indicate that flavor alone is sufficient for the vapors from an e-cigarette to alter gene expression in epithelial cells [19]. In another study, the exosomal miRNA contents of people who smoked various combinations of nicotine products were evaluated and compared against each other. People who exclusively smoked e-cigarettes had exosomal overexpression of several DNA-regulating miRNAs [5], including miR-100-5p, miR-125a-5p, miR-125b-5p, and miR-99a-5p, each of which had a role in DNA regulation and protection from cancer. The properties of these miRNAs and the functions they serve are summarized in Table 1. Upregulation of these miRNAs in exosomes implies an immune response to the introduction of regular nicotine doses, possibly including the increased risk of cancer that results from excessive e-cigarette usage.

Exosomes extracted from nicotine-treated macrophages increase the proliferation and migration of vascular smooth muscle cells (VSMCs) [39]. Not only that, but said exosomes also contain higher-than-normal levels of miR-21-3p, a miRNA involved in vascular injury and repair processes. Knockdown of miR-21-3p in VSMCs treated with these exosomes attenuated their negative outcomes. miR-21-3p binds to a phosphatase and tensin homologue (PTEN), the inhibition of which produced similar outcomes to exposure to exosomes from nicotine-treated macrophages, confirming that miR-21-3p is involved in the progression of nicotine-mediated atherosclerosis [40]. Not only do exosomes from nicotine-exposed macrophages promote atherosclerosis, but those who vape have significantly more exosomes in their blood [41].

## 4. Nicotine and Stroke

Stroke, the interruption of blood supply to the brain by ischemic or hemorrhagic means, is one of the more prevalent causes of death in the world. According to Tsao et al. [42], strokes accounted for approximately 1 in 6 deaths from cardiovascular disease in 2021, with a stroke occurring in an individual roughly every 40 s and a stroke-related death every 3 min and 14 s. Recent advances in treatment modalities have increased the number of patients who survive strokes, but many drugs fail to reach the CNS because of the BBB, which leads to many stroke survivors suffering cognitive impairment [43]. Ischemic stroke deprives the brain’s neurons of oxygen, causing severe cases of brain damage; a study by Jeffrey Saver [44] found that ischemic stroke can cause a loss of 1.9 million neurons every minute that the stroke goes untreated, equivalent to the brain aging 3.6 years every hour. The resulting brain damage is characterized by impaired speech, inability to perceive sensory input on one side of the body, visual field loss, and inability to comprehend or produce language. Deep enough infarctions can damage the thalamus, leading to cognitive impairment, hypersomnolence, hypoesthesia, and even ataxia [45].

Ischemic stroke can be predicted by assessing the variety of risk factors in a patient’s genetics and lifestyle. Risk factors for ischemic stroke can range from age and stress to poor diet and physical inactivity (NIH), but the risk factor that will be the primary focus for this review is the consumption of nicotine. In a survey by Appelros et al. [46], it was noted that nicotine use was one of a multitude of factors associated with different functional outcomes, including cognitive impairment and psychiatric disorders, in cases of ischemic stroke. Likewise, in a case study by Grzybowski et al. [47], it was found that chronic nicotine addiction is a risk factor for central retinal artery occlusion and cilioretinal artery occlusion, and several of the test subjects in this case study were also found to have focal ischemic cerebral changes—localized injuries caused by reduced blood flow to brain tissue. Another study by Pradhyumnan et al. [48] found that even brief e-cigarette vapor exposure over 2 weeks significantly impaired brain carbohydrate and lipid metabolism, exacerbated infarction, and impeded spatial learning and working memory. Nicotine is also thought to exacerbate ischemic stroke through multiple mechanisms. One mechanism is by altering the metabolic activity of neurons, which significantly reduces their survival rate post-ischemic stroke [49].

One other primary mechanism for stroke exacerbation by nicotine is through promoting inflammation. Another study found that both tobacco smoke and e-cigarette vapors, when mice were exposed to cigarette smoke containing 1.452 mg of nicotine an hour for 6–8 h a day for 2 weeks, increased nuclear factor erythroid-2 related factor (Nrf2) expression in mouse primary brain microvascular endothelial cells, which has been connected in the past to increased oxidative stress and inflammation and thus increased neurovascular damage in cases of ischemic stroke [50]. However, another study found that 30 mg/mL of tobacco smoke and e-cigarette vapor 6 times a day for 14 days could significantly reduce the expression of Nrf2, as well as upregulate ICAM-1, leading to significantly worsened ischemic brain injury [51]. Regardless of the mechanism, both studies show that nicotine alters the expression of proteins in the brain to promote a phenotype that significantly exacerbates brain damage as a result of ischemic stroke. In fact, in a data analysis study by Montes de Oca et al. [52], it was observed, through analysis of data from the Chilean Ministry of Health databases on hospital discharges and deaths, that a total ban on indoor smoking in Chile significantly reduced death rates from ischemic stroke as well as heart diseases, making it clear that nicotine affects increasing ischemic stroke rates and severity. Not only that, but nicotine use can also be a risk factor in ischemic stroke caused by other factors, as Ramphul et al. [53] found that among cannabis users suffering from acute ischemic stroke, 77.5% also smoked nicotine. More generally, a study by Paulson et al. [54] also found that nicotine increased the water content of mouse hippocampus slices during oxygen-glucose deprivation (OGD), which was theorized to be due to alterations of Na,K,2CL^−^ cotransporter NKCC. These results suggested that, through inflammation or otherwise, nicotine may be able to cause cerebral edema in stroke patients, explaining how nicotine makes ischemic stroke outcomes more severe.

Sex differences also cause nicotine to have different effects on the outcomes of ischemic stroke. In female rats, nicotine increases protein levels of caspase-1, apoptosis-associated speck-like protein containing a CARD (ASC), and pro-inflammatory cytokines interleukin (IL)-1β in the cerebral cortex [55]. These proteins significantly increased inflammasome activity and thus neuronal cell death. Similarly, the results of the aforementioned study by Pradhyumnan et al. [48] were found to be more significant in female mice than in males. One of the main reasons for these sex differences may be the synergistic effect of nicotine and estrogen, as it has been shown that nicotine directly hinders estrogen receptor-mediated phosphorylation of cAMP element binding protein [56]. This synergy was shown to significantly exacerbate post-ischemic damage when oral contraceptives containing 17β-estradiol were administered alongside nicotine.

Nicotine has also been shown to cause oxidative stress. In a Sprague-Dawley model of ischemic stroke, chronic nicotine exposure was found to increase concentrations of superoxides in the cerebral cortex by nearly 3.8-fold, along with significantly decreasing concentrations of UCP-2 (Uncoupling Protein 2, a protein that reduces reactive oxygen species (ROS) production in the mitochondria) in the cerebral cortex and MnSOD (a mitochondrial protein of critical importance in maintaining cellular ROS balance) in both the cerebral cortex and cerebral arteries [57]. However, this increase in superoxides may be due to increases in microglia activity due to altered exosome contents [58], as exosomal miR-125a-5p is shown to induce the oxygen free-radical-producing M1 phenotype of microglia [59] and is upregulated by E-Cigarette vapor exposure [5]. The aforementioned studies by Kaisar et al. [50] and Sifat et al. [51] also show this trend in nicotine increasing oxidative stress, and nicotine is so well-known as an oxidative stressor that Flores-Bellver et al. [60] used it as a means to simulate the effects of advanced aging. This oxidative stress, being able to simulate advanced aging, implies that cigarettes can also cause similar effects to those seen in conditions arising from advanced aging, such as increased tensity of the basilary artery wall.

Autophagy, the cellular process of digesting intracellular components, is typically an important tool for cells to recycle damaged organelles and misfolded proteins as well as dispose of pathogens [61]. However, in some cases of ischemic stroke, excessive autophagy can result in the breakdown of important cellular components, ultimately resulting in cell death [62]. Interestingly, it was also found that α7 nAchR activation increased both the autophagic signaling that prevents prion protein-mediated cytotoxicity and the autophagy of monocytes and microglia, the latter of which could prevent brain-damaging inflammation. However, these beneficial effects are largely dose-dependent; it was found by Xing et al. [63] that low doses of nicotine promoted autophagy and inhibited apoptosis in neonatal mouse cardiomyocytes, whereas high doses of nicotine inhibited autophagy and promoted apoptosis in those same cells [64].

In instances of stroke in a mouse model, nicotine significantly increases fatty acid metabolism and phosphatidylcholine accumulation without affecting enzyme levels, exacerbating existing brain damage via metabolic ATP deprivation [65]. Not only that, but mitochondrial complex IV activity was reduced by nicotine exposure, alongside reduced glucose-6-phosphate concentration and hexokinase activity, implying a decrease in glycolysis. The study also showed increased pyruvate and citrate concentrations and reduced concentrations of pyruvate kinase isoform M2 and lactic acid dehydrogenase, implying alterations to the Krebs cycle to reduce aerobic respiratory activity [66]. Nicotine exposure also significantly increased levels of histidine metabolites in the cortex, as well as reducing levels of N-methyl-GABA and glutamic acid decarboxylase, leading to further reduced post-ischemic perfusion [67]. Furthermore, a study by Sifat et al. [68] showed that nicotine exposure significantly decreased glycolysis, glucose uptake, and expression of glucose transport proteins GLUT1 and GLUT3 in the brains of a mouse model of ischemic brain injury, leading to glucose deprivation and more severe stroke outcomes. The reduced expression and function of GLUT1 was corroborated by a study by Shah et al. [69], and Xu et al. [70] shows that GLUT1 can be downregulated through PPARα hyperactivation by exosomal IL-8, which may be upregulated by nicotine use (see Table 1). These results show that nicotine exposure can drastically alter metabolic activity and mitochondrial function, leading to further exacerbated brain damage in cases of ischemic stroke.

In some models of ischemic stroke, nicotine also damages the blood–brain barrier (BBB) and the flow of blood throughout the brain as well. A study by Kumar et al. [71] found that nicotine withdrawal increased BBB permeability in the prefrontal cortex (PFC) of female mice, and depletion of microglia reversed this effect. Another study found that both combustible cigarettes and e-cigarettes significantly altered the transcriptional profile of brain microvessel endothelial cells, significantly reducing cortical expression levels of tight-junction protein Occludin as well as glucose transporter Glut1 [72]. These disruptions of protein expression significantly increase BBB permeability, which can promote inflammation in addition to allowing further infiltration of nicotine and potentially other harmful compounds.

In a similar sense, nicotine exposure leads to altered gene expression that may yield phenotypes that are not only more likely to suffer an ischemic stroke but also more likely to encounter more severe complications from an ischemic stroke. For example, in one experiment by Hu et al. [73], mouse aortic smooth muscle cells were pretreated either with or without Epoxyeicosatrienoic acids (EETs), followed by nicotine with or without treatment with a cocktail of Selisistat, 14,15-EEZE, and 11,12-DHET. The administration of these soluble epoxide hydrolases preserved Sirtuin 1 expression, leading to reduced arterial stiffness. These in vitro findings align with observations from earlier in vivo findings in the same study showing a correlation between reduced EET production and increased arterial stiffness in Ephx2^−/−^ mice compared to wild-type controls.

Given that nicotine exposure led to reduced SIRT1 expression, it is not too big a leap in logic to conclude that nicotine exposure could exacerbate arterial stiffening, leading to worse outcomes. Furthermore, another study found that cigarette smoke extracts from both ultra-low nicotine products and nicotine products with standard amounts of nicotine (AKA “Full Flavor”) significantly upregulated expression of NFκB-p65, a potential activator of oxidative and inflammatory stress pathways, alongside pro-inflammatory IL-6, by immortalized human BBB endothelial cells [74]. This not only indicates that an immune-like reaction to nicotine is taking place, but it also shows that oxidative stress is exacerbated down to the genetic level, providing further evidence of how nicotine use exacerbates stroke.

Paradoxically, some studies have shown that nicotine use can somehow increase the likelihood of good outcomes in ischemic stroke. The exact mechanisms behind this phenomenon are unclear, but this oddity is so well-known that it has earned itself an official name: the “Smoker’s Paradox.” However, a study by Hussein et al. [75] found that not only was the Smoker’s Paradox not found in ischemic stroke patients treated with intravenous thrombolysis, but also that ischemic stroke patients who smoked nicotine products had their first ischemic stroke 11 years younger than those who did not smoke, These results were corroborated by a meta-analysis by Zhang et al. [76], it was found that there was no significant correlation between nicotine smoking and good functional outcomes of ischemic stroke. Several mechanisms for the “Smoker’s Paradox” have been proposed, including CYP1A2 and CYP2C19 enzyme hyperactivation acting on the anti-platelet effect of the heart medication Clopidogrel, increased P2Y12 receptor expression increasing the anti-platelet effect of Clopidogrel, increased genetic sensitivity to anti-platelet medication, and genetic polymorphisms that cause CYP1A2 to lose function unless nicotine is present [77]. Regardless, the meta-analysis results suggest that any indication that nicotine smoking may improve stroke outcomes is the result of genetic and medication factors that are only tangentially related, rather than a genuine effect of nicotine use, emphasizing the need for prevention therapy to combat the increasing incidence of smoking-related health issues.

## 5. Mechanisms Underlying Nicotine’s Impact on Ischemic Stroke Through Exosomes

Considering that nicotine increases the levels of miR-21-3p in circulating exosomes [40] and that high miR-21-3p activity is implicated in the severity of ischemic brain injury outcomes [78], it is not a large logical leap to deduce that exosomes altered by nicotine increase the probability of ischemic stroke. Although the correlation could not be found in patients who smoked [79], it is also worth noting that in ischemic stroke patients who did not smoke, the patients who had an ischemic stroke had lower levels of IGF2 circulating in their plasma exosomes compared to patients who did not have a stroke.

One of the more significant trends in ischemic stroke research is the observation of similarities in the exosome content between strokes and certain types of cancer that can be caused by smoking. For example, significant upregulation of LGALS3 is prevalent in brain damage sustained as a result of ischemic stroke [80]. Additionally, scavenger receptor, CD36, and pattern-recognition receptor, CD14, expression were also significantly upregulated in stroke model exosomes [81]. Human macrophages exposed to nicotine in vivo showed increased expression of CD36 and CD14 [82], and since exosomes can pass through the body into the bloodstream [3,4] and cross the BBB [2], it stands to reason that nicotine can exacerbate the severity of a stroke by increasing expression and delivery of CD36 through macrophage exosomes.

In some cases, the effects of nicotine on stroke outcomes are not apparent in the immediate smoker, but rather they are a genetic fault that the offspring must carry. Other times, the story of how nicotine affects stroke probability is far more complicated than a clear “if X, then Y, else Z” relationship between nicotine use, protein levels, and their effects. The synaptic factor PSD95 was one of the proteins whose expression was reduced in cultured post-stroke depression (PSD) hippocampal cells exposed to exosomes from mouse stroke models [83]. Although nicotine exposure in adult mice did not provide any significant alterations in PSD95 expression [84], the offspring of mice that were exposed to nicotine while pregnant showed increased PSD95 expression in the microglia of the hippocampus [85]. At first, this is contrary to established research suggesting that nicotine exposure increased the probability of stroke [86]. However, another paper showed that increased formation of an NMDAR/nNOS/PSD95 ternary complex led to neurotoxic effects in the offspring of nicotine-exposed rats [87]. As well, some of the research that appears to yield no results may, in fact, hint at where to look.

This theory is further enforced by the findings that exosomes can also spread complications from one subject to another. Exosomes from stroke-afflicted rats can exacerbate stroke-related complications in recovering rats. Clinically, 23–67.74% of stroke survivors become depressed after surviving their stroke [88]. In animal models, PSD is linked to increased levels of p75NTR and proBDNF alongside decreased levels of synapse-associated protein [83]. To test these effects, exosomes were isolated from the hippocampus of mouse stroke models, and these exosomes were administered to cells cultured under OGD/R and PSD conditions. The exosome-exposed PSD cells had lower cell viability, increased levels of pro-inflammatory p75NTR and proBDNF, and reduced levels of PSD95 and synaptotagmin compared to control cell cultures and even to the non-exosome-exposed PSD group. Thus, both when healing and when harming, exosome contents have a large influence on outcomes during and after cases of ischemic stroke, which further emphasizes how very important the study of exosomes in ischemic stroke is, especially when exosome-altering factors such as nicotine are involved.

Restricted Cubic Spline (RCS) analysis also found a nonlinear correlation between plasma exosomal *circZNF609* expression levels and the risk of ischemic stroke development in human patients [79]. Patients with ischemic stroke also had lower levels of IGF2, and although no linear correlation could be found between plasma exosomal IGF2 levels and ischemic stroke development, the difference in expression levels alone implied a protective effect of IGF2 against ischemic stroke development. However, this connection was only statistically significant in patients who did not smoke; in the smoker subgroup, levels of plasma exosomal IGF2 were not significantly different.

Nicotine has been shown in the past to alter the function of cells in the neurovascular unit, the interface of neurons and the endothelial cells of the blood vessel. For example, in an in vitro experiment, doses of nicotine up to 50 ng/mL significantly impaired the viability of mouse C8D1A astrocytes after 12 and 18 h, and even doses of nicotine as small as 10 ng/mL nearly doubled rates of astrocyte apoptosis after 12 h. Nicotine also increased the expression of IL-6, IFNγ, and TNFα in a dose-dependent manner, increasing over 48 h, suggesting that nicotine can drive A1 polarization of astrocytes [89]. Considering that high levels of IL-6 contribute to the brain damage seen in ischemic stroke [78], the consistent evidence that IL-6 expression is increased by e-cigarette use reinforces the theory that e-cigarette use increases the chance of ischemic stroke. Even if the overarching knowledge is largely inconclusive, there is more often than not something conclusive. This is not to say, however, that all results from background research into stroke-related proteins affected by nicotine yielded corroborative evidence. Several proteins and pathways, such as JAK-STAT3 signaling, TLR2 expression, and STAT5 signaling, were reduced by exposure to nicotine [80,90]. However, exosomes from stroke models showed that these pathways were enriched in cases of ischemic stroke [91]. While correlation does not necessarily equal causation, it is still important to keep an eye on trends and patterns in the expression of genes and proteins, especially the effects of various drugs on those proteins, in order to discern what is known for certain and what could be studied further. Thus, even when the premise (or focus) of the paper is that nicotine increases the prevalence of stroke, it is important to take note of instances where nicotine seems to decrease the prevalence of stroke.

However, it is also important to consider instances where nicotine appears to have the opposite effect: a neuroprotective effect on ischemic stroke, rather than an exacerbating effect. One study by Ouro et al. [92] found that inhibiting AMPK, especially by systemic administration of CMV-AMPKα2-DN-containing small EVs, reduced brain lesion volume, edema volume, and improved neurological recovery from stroke, as evaluated by the Bederson score, in rat and mouse models after 7 days post-treatment. This inhibition could be achieved by administering small extracellular vesicles (i.e., exosomes) containing the dominant negative isoform of AMPKα2 (AMPKα2-DN sEVs), but curiously, it was also found that this effect could be achieved by administration of nicotine. In general, taking all of the evidence and prior research into account, it appears to be likely that nicotine-mediated alterations in neuronal exosome content have an effect on the progression of stroke in nicotine users.

## 6. Potential Biomarker and Therapeutic Role of Exosomes in Stroke with Nicotine Exposure

The ability of exosomes to cross the BBB also makes them reliable biomarkers for ischemic brain damage. Exosomes from neurons have a unique protein signature, including L1 cellular adhesion molecule (L1CAM) and glutamate-aspartate transporter (GLAST), and a significant amount of circRNAs were also differentially expressed in cases of viral and bacterial infections in the CNS [93]. The cited study had extracted neuronal exosomes from the blood plasma, showing that cases of neuronal dysfunction can cause neuronal exosomes to leak into the bloodstream. Meanwhile, a study by Perets et al. found that exosomes derived from mesenchymal stem cells in bone marrow (MSC-Exos) specifically target and accumulate in pathogenically relevant areas of murine model brains, in conditions ranging from autism to ischemic strokes; in this study, MSC-Exos were administered intranasally, implying that they could cross the BBB through the olfactory nerve [94]. Likewise, a study by Kojima et al. found that subcutaneously injected designer exosomes could deliver therapeutic cargo for Parkinson’s disease treatment into the brain, significantly increasing neuron survival rates [95]. These two studies both indicate that exosomes can cross the BBB and enter the CNS.

Exosomes are involved in such a wide variety of conditions that medicinal research designed to find treatments for one condition yields results that can shed light on another. For instance, when nicotine-treated rats were administered Crocin (a key carotenoid component of saffron), levels of hippocampal TNF-α, IL-1β, BAX, and Beclin 1 were significantly reduced compared to nicotine-treated rats that were not given Crocin. Not only that, but motor activity and behavioral changes observed in nicotine-treated rats were not observed in the rats that were given both nicotine and Crocin, and Crocin treatments increased the activity of mitochondrial complex enzymes [96]. These results suggest that upregulation of TNF-α, IL-1β, Bax, and Beclin 1 significantly increased neuronal oxidative stress, leading to defects in the CNS that yielded anxiety-like and motor-problem phenotypes. Increased production of TNF-α and IL-1β is responsible for the brain damage caused by ischemic stroke [78], upregulation of Bax led to increased apoptosis and thus neuronal damage [97], and exosomes derived from human neuronal stem cells (hNSC-Exos) upregulated Beclin to promote neural autophagy [81], so this is further evidence not only that nicotine can promote strokes, but also that exosomes derived from healthy brain cells can pave the way for more effective treatment of neuropathies, including nicotine use disorder.

Exosomes have shown promise as a treatment for ischemic stroke, since they can cross the BBB and deliver proteins and miRNAs to reduce the infiltration of various types of leukocytes into brain tissue [98,99]. MMP-9 inhibition by administration of exosomes from healthy rats helped reduce leakage of the BBB in ischemic stroke models [12], which helped improve outcomes 24 h after stroke. Meanwhile, nicotine increases MMP-9 expression in the hippocampus, leading to the induction of nicotine CPP [11]. If a drug increases MMP-9 expression in one part of the brain, it can likely do similar in other parts of the brain, including the BBB, leading to degradation of tight-junction proteins. This degradation leads in turn to leakage of the BBB and thus worse stroke outcomes.

Exosomes containing compounds such as nicotinamide phosphoribosyltransferase (Nampt) play an important role in the regulation of autophagy and preventing cell death in ischemic stroke [97]. The NAMPT gene, meanwhile, is significantly downregulated in cases of nicotine toxicity in nucleus pulposus (the gelatinous core of the intervertebral discs) cells [100]. Nampt has protective effects against senescence in general, so the theory that nicotine downregulates Nampt expression corroborates nicotine’s use as an oxidative stressor to simulate aging [60]. In addition, Pro-inflammatory p75NTR expression was increased in the lungs of nicotine-exposed rats [101], and nicotine was also found to impair proBDNF proteolysis in multiple brain regions in adolescent mice [102]. Likewise, cultured PSD cells exposed to exosomes from mouse stroke models showed increased expression of p75NTR and proBDNF [83], leading to reduced cell viability in PSD models. Taken together, nicotine does seem to lead to reduced cellular viability in PSD, increasing the prevalence of uncontrolled apoptosis and cancer. Nicotine has also been found in the past to aggravate the postischemic inflammatory response in the brain [103], and that aggravation includes overexpression of ICAM1, IL-1β, and TNF-α. Considering that exosome analysis has found that ICAM1 [81], IL-1β, and TNF-α [78] are increased in ischemic brain injury, this observation lines up well with established research, proving the efficacy of exosomes as a diagnostic tool. More importantly, these findings also show that nicotine yields an expression phenotype similar to exosomes from ischemic brain injury, further implicating nicotine use as a risk factor for severe stroke outcomes.

Exosome therapy efficiency can be improved by optimizing isolation techniques and engineering exosomes with particular mechanisms of uptake tailored to the target cell [2]. However, there are still multiple barriers to exosome therapy becoming commonplace, including unknown cargo capacity and half-life, unclear considerations for dosage and biodistribution, and unknown kinetics of uptake by target cells, as well as difficulties with targeting specific organs and tissues. Many past studies have experimented with utilizing exosomes as a drug delivery mechanism. Such experiments show promising results, although the treatments are still experimental. For example, a preliminary study by Haney et al. showed that catalase-loaded exosomes could be readily taken up by neuronal cells in vivo, as well as having a protective effect against Parkinson’s Disease [104]. Considering that Parkinson’s is a disease that is linked with altered dopaminergic signaling, it is not unreasonable to assume that exosomes could also be utilized to treat other illnesses of the brain and nervous system, including substance use disorders.

One of the subjects of research in drug treatments for ischemic strokes is nicotine. Unfortunately, even when its purported beneficial effects are the main focus of a study, there are often multiple side effects or caveats that reduce the potential benefits. For instance, nicotine has been found to inhibit the cleavage of caspase-3 in a mouse model of Parkinson’s disease [105]. While this is largely good news, there are conditions in which increased cleavage of caspase-3 can be beneficial. For example, increased caspase-3 cleavage can improve ischemic stroke outcomes by inhibiting endothelial cell apoptosis [61,81]. Thus, inhibition of caspase-3 cleavage can lead to worse stroke outcomes. Similarly, nicotine has also been shown to upregulate Bcl-2 and downregulate Bax [106,107], both of which have been found to prevent neuronal death in cases of neurodegenerative diseases such as Parkinson’s, Alzheimer’s, and ischemic stroke [97]. Despite this neuroprotective property, the other, unhealthy effects of nicotine may override the benefits of modulation of the Bcl-2/Bax ratio, and the prevention of apoptosis may also enable the development of cancer in nicotine-exposed cells. Furthering this point about nicotine causing more problems than it solves, LC3B is upregulated by hNSC-Exos in order to promote mitophagy and reduce oxidative stress [81]; meanwhile, pretreatment of rats with an autophagy-inhibiting drug led to accumulation of LC3B-II and counteracted nicotine-mediated decreases in mtDNA copy number, although analysis of human neuroblastoma SH-Sy5y cells found that nicotine treatment also induced several mitochondrial defects [108]. Taking this together, it appears that LC3B upregulation in nerve cells is a side effect of damage to the mitochondrial DNA, leading to increased autophagic activity.

Another thing to consider is that the whole point of PINK1 and Parkin upregulation by hNSC-Exos is to prevent defective mitochondria from causing the cell oxidative stress [109]. Considering that nicotine is so effective at causing oxidative stress that it can be used to simulate the effects of aging [60], it is a distinct possibility that hNSC-Exos could be used to protect from nicotine-related stroke complications. The role of exosomes in both exacerbating, diagnosing, and treating ischemic stroke outcomes in nicotine users, as was discussed thoroughly in this review, is not to be understated.

Nicotine-selective protection of dopaminergic neurons in the substantia nigra in *C. elegans* worms was also due to the confluence of specific D3-receptor expression and their vulnerability to mitochondrial stress, the latter of which is mitigated by increased mitochondrial quality control due to PINK1 activation [110,111]. As a side effect of this inhibitory effect, smoking tobacco reduces the prevalence of Parkinson’s disease. However, in other cells—particularly the ventricular myocytes of neonatal rats—exposure to nicotine led to increased mitochondria-derived superoxide production and impaired PINK1/Parkin-mediated mitophagic flux [112]. Considering that hNSC-Exos upregulated both PINK1 and Parkin to promote mitophagy in order to reduce oxidative stress [109], the question of whether nicotine activates or impairs PINK1 and Parkin in the cells of the central nervous system remains a pressing issue to address in the future.

## 7. Future Directions

In general, nicotine and ischemic stroke both have clear influences on the contents of exosomes produced by neurons and other brain cells, but this review mainly focuses on neuronal exosome production. A future study, or even a future review, would benefit greatly from investigating the contents of exosomes from other cells in the brain. Astrocytes, for example, play a crucial role in regulating the BBB, and microglia serve as immune cells that modulate inflammatory responses and guard against infection. Since some of the observed results were also involved in cells outside of the CNS, such as cardiomyocytes and breast carcinomas [80,113,114], examining the effects of nicotine on the brain cells’ levels of specific proteins and miRNAs remains a pressing matter to examine further.

In fact, a large body of research already exists on the influence that exosomes have on astrocytes and microglia. For example, Guo et al. implied that administration of microglial exosomes after microglia deletion increased astrocyte inflammation and Parkinson’s disease pathogenesis in a mouse model [115]. Likewise, in a study by Deng et al., it was found that administration of exosomes from human astrocytes reduced the presence of Aβ42 oligomers in a mouse model of Alzheimer’s disease [116]. Lastly, it has been found that astrocyte-derived exosomes have the potential to ameliorate neural inflammation in cases of traumatic brain injury and, connecting to the present review, induce autophagy to ameliorate acute ischemic stroke injury [117,118]. This pre-existing knowledge base provides a high-quality starting point for future studies into how drugs such as nicotine can alter exosome contents and influence the function of cells in the brain.

Although many proteins that were discussed in this review were found to be altered in both strokes and nicotine and could be regulated by exosomes, there were still many that had no papers written on their relation at all. Experiments on the effects that nicotine has on the expression of Shoc2, miR-190-b, and miR-129-1-3p, for example, may prove to be a large boon to the advancement of not only stroke research but the formation of exosome-based treatments as well. That being said, correlation does not necessarily imply causation; thus, to confirm that the changes in protein and miRNA expression in cells and their exosomes yield similar results, it may be prudent to form dedicated research examining these connections. As a beneficial effect, these experiments provide the perfect opportunity to investigate treatments that target affected proteins/miRNA as a means to ameliorate the deleterious effects of both stroke and nicotine addiction.

One other factor to note in this review is that it focuses heavily on the correlation between nicotine use, changes in exosome contents, and stroke pathogenesis. However, this is still heavily speculative, as there is little direct evidence for a definitive causal connection between the three. Examining the effects of nicotine use on stroke pathology with an emphasis on exosomes will likely require its own dedicated study in the future. However, this also yields its own difficulties, as there remain several challenging issues to handle when conducting research on EVs. For example, there is no pre-defined standard for how EV research should be conducted, how exosomes should be purified, and how EVs should be characterized and administered [119]. Purifying exosomes in particular is exceptionally difficult because of their small size, and so there will likely be at least some microvesicle contamination unless special antibodies conjugated to magnetic beads are used in a process called “Affinity Capture.” [120]. Lastly, despite one of the primary purported draws of EV research being that EVs can be used to target specific body regions for drug delivery, it is difficult to control where the EVs go when they are administered, compounded by how most EV studies administer EVs systemically [119]. Even when EVs are being administered to specific brain regions, however, their propensity to cross the blood–brain barrier means that they could cause side effects in seemingly unrelated regions of the brain and body [121]. Further testing is necessary to determine a safe and reliable means to administer exosomes as a medical tool, and research organizations and policymakers need to determine specific standards for how future research into EVs is to be conducted.

## Figures and Tables

**Table 1:** Summary of findings on different types of nicotine products on microRNA expression. Unless otherwise noted, all differences in miRNA expression were determined in plasma exosomes relative to non-smokers.

Exosome Content	Type of Nicotine Product			Function	Potential Impact on the Brain
	Tobacco	Waterpipe	E-Cig		
miR-100-5p	Overexpressed [14,20]	No data [14]	Overexpressed [5,14]	Involved in DNA damage response, also implicated in smoking initiation and educational attainment; underexpression contributes to tumor malignancy (Genecards)	Overexpression of miR-100-5p was found in a late-stage mouse model of Alzheimer’s disease [21] as well as in patients with ischemic stroke [22].
miR-125a-5p	Underexpressed [23]	No data [14]	Overexpressed [5]	In a rat model of epilepsy, miR-125a-5p overexpression inhibited inflammatory factor expression and alleviated seizures by downregulating calmodulin-dependent protein kinase IV (CAMK4) [24]	CAMK4 is important for the long-term depression pathway (LTD), and excessive inhibition of CAMK4 in the cerebellum is associated with impaired learning and memory [25]
miR-125b-5p	Overexpressed [14]	No data [14]	Overexpressed [5]	Involved in glioblastoma (Genecards), downregulates chemokine receptor CCR7 and upregulates monocyte trafficking in cases of atherosclerosis [26]	MMP-13 reductions impair angiogenesis and significantly reduce newborn neuroblasts in cortical peri-infarct, reducing repair from ischemic stroke [27]. Overexpression of miR-125b-5p was also found in patients with ischemic stroke [22].
miR-29b-3p	Overexpressed [20]	Underexpressed [14]	Underexpressed [14]	Inhibitor of beta-secretase 1 (BACE1), an enzyme implicated in beta-amyloid production in Alzheimer’s disease (Genecards). BACE1 deletion helps prevent memory loss in a mouse model of Alzheimer’s [28]	Underexpression of miR-29b-3p could increase BACE1 activity, serving to exacerbate Alzheimer’s in chronic smokers.
miR-365a-3	No data [14]	No data [14]	Overexpressed [14]	miR-365a-3p directly inhibits MyD88 expression and downstream NF-κB activation [29]	MyD88 inhibition may result in reduced memory/motor deficits, decreased pro-inflammatory factors, microglial inactivation, synaptic protein retention, and reduced neuronal apoptosis [29]. However, a mouse study connected MyD88 deficiency to decreased locomotor activity, increased anxiety-like behavior, and potential cognitive inflexibility [30].
miR-365b-3	Overexpressed [23]	No data [14]	Overexpressed [14]	Reduced expression in Alzheimer’s Disease. Predicted to regulate APOM and MAPT [31]	APOM is expressed in endothelial cells comprising the BBB [32], whereas MAPT SNPs were significantly associated with older relative brain age and signs of impaired fluid intelligence [33]. However, no significant concrete link between nicotine intake and either of these genes could be found.
miR-99a-5p	Underexpressed [23]	No data [14]	Overexpressed [5]	In human cases of ischemic stroke, miR-99a silencing by circCNOT6L served to attenuate cell apoptosis and alleviate stroke injury [34]	MiR-99a overexpression in a case of ischemic stroke could lead to increased cell apoptotic signals through the overexpression of serine protease inhibitor SERPINE1 [34]. This would increase the rate of cell apoptosis and exacerbate brain damage caused by ischemic stroke.
Let-7a-5p	No data [14]	Overexpressed [14]	Overexpressed [14]	Abundance of Let-7a-5p is associated with Reversible Cerebral Vasoconstriction Syndrome, which could impact BBB health [35]. Let-7a-5p was also found to be upregulated in a cultured human microglia model of SARS-CoV-2 Spike-1 mediated neuroinflammation [36]	Let-7a-5p’s implicated role in neuroinflammation could foretell an increased likelihood of worse ischemic stroke outcomes in patients who use e-cigarettes.
Let-7i-5p	Overexpressed [14]	Overexpressed [14]	No data [14]	Mouse let-7i-5p was found to be upregulated in a mouse model of Alzheimer’s disease [37] and was also elevated following epileptic seizure [38]	Let-7i-5p upregulation is associated with worse functional outcomes in multiple neurological conditions, so it would also yield worse functional outcomes in ischemic stroke. However, this link is mainly speculation at this phase of research.

## Data Availability

No new data were created or analyzed in this study. Data sharing is not applicable to this article.
